# “Liu-Liang-Chung” syndrome with multiple congenital anomalies and the distinctive craniofacial features caused by dominant *ZEB2* gene gain mutation

**DOI:** 10.1186/s12887-023-04314-5

**Published:** 2023-09-21

**Authors:** Wei-Liang Liu, Fang Li, Wei Chen, Lu Liu, Hai-jian Cheng, Zhi-Xu He, Rong Ai

**Affiliations:** 1 Department of Pediatrics, Affiliated Hospital of Guizhou Medical University, Guiyang, 550004 China; 2 Department of Ophthalmology, Affiliated Hospital of Guizhou Medical University, Guiyang, 550004 China; 3 Beijing Kangso Medical Laboratory Co., Ltd, Beijing, 100195 China

**Keywords:** Liu-Liang-Chung syndrome, *ZEB2*, Increase of gene dosage, 2q22, Distinctive craniofacial features

## Abstract

**Background:**

Contiguous gene gain syndrome including entire *ZEB2* may be a novel syndrome. In the past, there were no easily distinct and recognizable features as a guide for precise clinical and genetic diagnosis of the syndrome.

**Case presentation:**

We report a novel case with the syndrome with a novel *de novo* 22.16 Mb duplication at 2q21.2-q24.1. The syndrome is characterized by multiple anomalies including the same typical craniofacial phenotype that is entirely different from Mowat–Wilson syndrome (MWS), and other quite similar features of MWS consisting of development delay, congenital heart disease, abdominal abnormalities, urogenital abnormalities, behavioral problems and so on, in which the distinctive craniofacial features can be more easily recognized.

**Conclusions:**

Contiguous gene gain syndrome including entire *ZEB2* characterized with similar multiple congenital anomalies of MWS and the distinctive craniofacial features is mainly caused by large 2q22 repeats including *ZEB2* leading to dominant singe *ZEB2* gene gain mutation, which is recommended to be named “Liu-Liang-Chung” syndrome. We diagnose this novel syndrome to distinguish it from MWS. Some variable additional features in the syndrome including remarkable growth and development retardation and protruding ears were recognized for the first time.

**Supplementary Information:**

The online version contains supplementary material available at 10.1186/s12887-023-04314-5.

## Background

Mowat–Wilson syndrome (MWS, OMIM: 235,730) was first described in 1998 in six patients who presented characteristic facial features, Hirschsprung disease, microcephaly, mental retardation [1]. A lot of additional features have been reported subsequently, including variable multiple congenital anomalies, epilepsy, behavior problems and so on [2–6]. Heterozygous gene mutations in the *ZEB2* gene located on 2q22 or deletion in the chromosome 2q22–2q23 have been shown to cause MWS [1, 7, 8]. However, the increase of entire *ZEB2* exonic copy number has not been reported in MWS. To address the phenotypic spectrum of the contiguous gene gain syndrome including entire *ZEB2* for precise cytogenetic type-phenotype correlation research in future, we report a novel case with the syndrome with a novel 2q21.2-q24.1 duplication including entire *ZEB2*. Recently, A few individuals with interstitial chromosome gain of 2q22.3 including the entire *ZEB2* gene have been identified by Liang et al. and Chung et al. [9, 10]. These patients may have most of the features of MWS such as development retardation, congenital heart disease, chronic constipation. However, they present with distinctive craniofacial features, which do not resemble classical facial features of MWS.

## Case presentation

The patient as a novel case is a 2.6-year-old Chinese girl and is the only child of the family. She was born to healthy parents who are non-consanguineous. Her father has single palmer creases in both hands. During pregnancy, the mother had a history of upper respiratory tract infection in the first month. Though feeling less fetal movement, she had no prenatal examination. She was born at 41^+2^ weeks (weight, 2.7 kg), with oligohydramnios, by caesarean section due to social factors. The Apgar scores were normal. The patient was referred to our hospital at the age of 2.6 years because of development delay. At 1 year of age she was able to recognize people. She could sit at 1.5 years of age. She still could not climb and stand now. Expressive language was markedly delayed with first word at 2.5 years. Thought she is able to make involuntary sound of only 2 words (mama, papa) now, her language’s comprehension skills are poor. She has chronic constipation after birth, about once every 10 days, without feeding difficulty and vomiting.

**Fig. 1:**
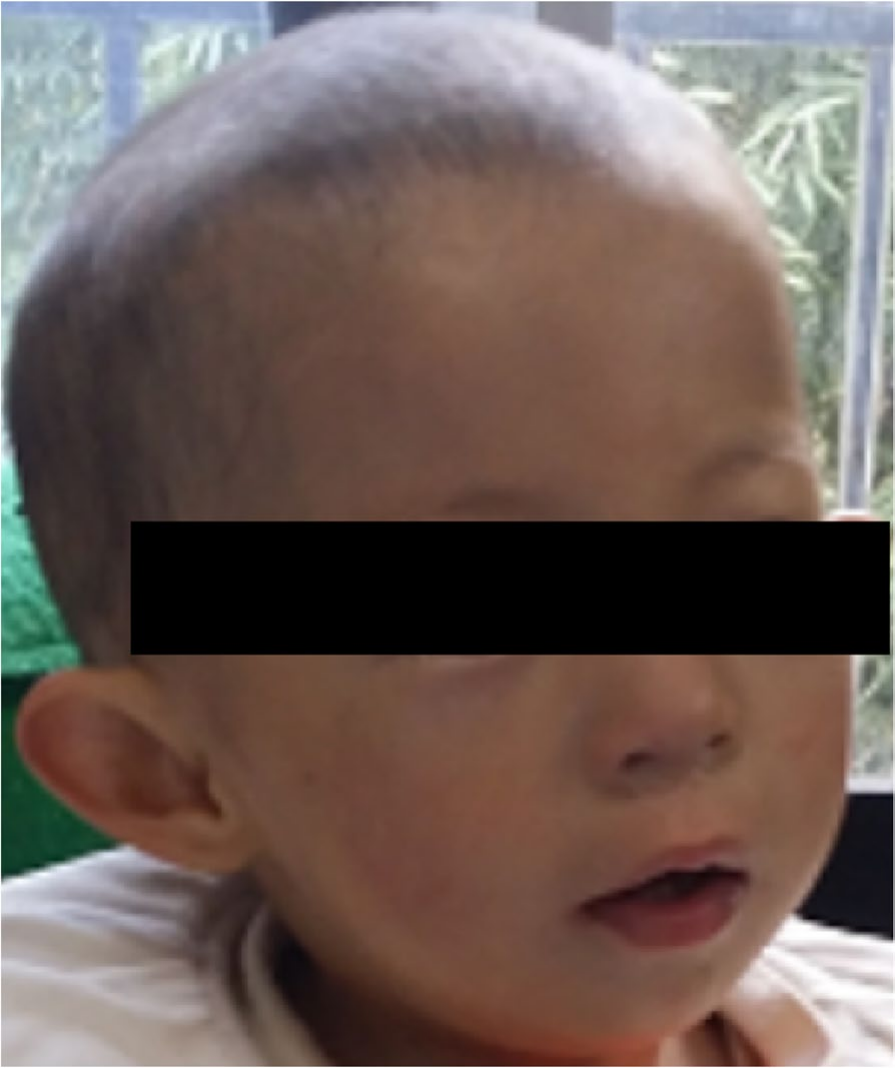
A photograph of the present case

Clinical examination at admission revealed a weight of 7 kg (< 3th centile), a length of 78 cm (< 3th centile) and a head circumference of 45.5 cm (< 3th centile), indicating obvious physical retardation. Her most distinctive craniofacial features included slight raising eyebrow’s eyes (slight ptosis with compensatory elevated eyebrows), small eyes, slightly swollen upper eyelids, hypertelorism, sparseness of the whole eyebrows and hair, low nasal bridge, small nose, shallow philtrum, small and open mouth, thin upper lip, high arched palate, intermittent sparse teeth, micrognathia, low-set ears, protruding ears, circular arc helix, dysplasia of auricle, crus of helix and earlobe, no antihelix and crus of antihelix, flat forehead and face, and scaphocephaly (Fig. 1). Besides, she never presented with prominent chin, uplifted earlobes, rounded nasal tip, prominent columella, medial flaring of the eyebrows, large eyebrows, sparse eyebrows in the middle, nystagmus, strabismus, and epicanthal folds of craniofacial features of classical MWS. Also, no significant abnormalities on vision, hearing, muscular tension, navel and four extremities including fingernails and toenails were noticed. The patient was easily to be irritated and prone to crying. However, she had no behavior of stereotype, frequent smiling and seizure. The patient has bilateral indirect inguinal hernia. Electroencephalogram (EEG) and B-ultrasound of liver, gallbladder, spleen, pancreas and kidney were normal. Doppler echocardiogram revealed an atrial septal defect (oval fossa type). Abdominal radiology of barium enema showed dolichosigmoid (Supplemental Fig. 1a). Brain magnetic resonance imaging (MRI) not only showed that the volume of frontal lobe was small but also showed that encephalomalacia was around the left ventricle and delayed myelination was in the posterior horn of both lateral ventricles (Supplemental Fig. 1b).

We analyzed clinical data and collected DNA samples from the child and her clinically unaffected parents. The present study was approved by the Ethics Committees of Affiliated Hospital of Guizhou Medical University (approval no. 20,101,124). Written informed consent and permission have been obtained from the parents of the patient for publication of the study and accompanying images.

We used xGen Exome Research Panel v1.0 kit (Integrated DNA Technologies, Skokie, Illinois, USA) for next‑generation sequencing (NGS) according to the manufacturer’s protocol. Chromosomal copy-number variations (CNVs) were tested based on NGS by Burrows-Wheeler Aligner (BWA v.0.6.2). Human reference genome used was from GRCH37/hg19. The results of CNVs were further verified by using fluorescence quantitative PCR (fq-PCR). Four pairs of primers available on request were designed to amplify specific gene sequences including the *NCKAP5*, *LRP1B*, *KCNJ3*, *ZEB2* genes, respectively. The first three genes lie in the proximal, central and distal ends of the region of the variant chromosome, respectively. In the region, important *ZEB2* gene was also amplified. A normal copy number DNA sample as a control and test samples for fq-PCR (7300Plus Real-Time PCR System) were detected by the UltraSYBR Mixture (High ROX). Data analysis of PCR products was performed by Real-Time PCR Software V2.4. Gene copy numbers were assessed by the ΔΔCt method according to the manufacturer’s recommendations. We present a literature review of all cases with detailed clinical data of the contiguous gene gain syndrome including the entire *ZEB2*.

A novel *de novo* 22.16 Mb duplication at 2q21.2-q24.1 including the striking *ZEB2* and other 41 genes in OMIM-listed database, (133,460,001- 155,620,000) x3, was identified by NGS-based detection of CNVs (Supplemental Tables 1, Supplemental Fig. 2). The results of CNV have been further validated using fq-PCR (Supplemental Fig. 3). This CNV was not found in her parents. Study of the family constellation indicated that the CNV arose *de novo*.

The distinctive craniofacial features of our patient and previously reported cases [9, 10] with the contiguous gene gain syndrome including entire *ZEB2* are raising eyebrow’s eyes, small eyes, slightly swollen upper eyelids, hypertelorism, sparseness of the whole eyebrows, low nasal bridge, small nose, shallow philtrum, small mouth, thin upper lip, micrognathia, low-set ears, flat forehead and face, and scaphocephaly. However, the characteristic craniofacial features of MWS are not found in the contiguous gene gain syndrome including entire *ZEB2*. We first proposed these similar craniofacial features, through which the disease can be better diagnosed. In addition, these patients with the contiguous gene gain syndrome including entire *ZEB2* may have neurodevelopment retardation (3/3), congenital heart diseases (3/3), constipation (2/3), dental anomalies (2/3), behavioral problems (2/3), inguinal hernia (1/3), and urogenital anomalies (1/3), which are also common features in MWS patients. Additional remarkable physical retardation and ear deformity (protruding ears) in our case were reported in the syndrome. Major clinical features in two previously reported individuals and our case with the contiguous gene gain syndrome including entire *ZEB2* by a cytogenetic diagnosis are listed in Table 1.

**Table 1:** Major clinical features in reported individuals with Liu-Liang-Chung syndrome

Clinical features	Case1 [9]	Case2 [10]	Our case
**Craniofacial features**			
Raising eyebrow’s eyes	+	+	+
Small eyes	+	+	+
Slightly swollen upper eyelids	+	+	+
Hypertelorism	+	+	+
Sparseness of the whole eyebrows	+	+	+
Low nasal bridge	+	+	+
A small nose	+	+	+
Shallow philtrum	+	+	+
Small mouth	+	+	+
Micrognathia	+	+	+
Low-set ears	+	+	+
Dysplasia of auricle and earlobe	+	-	+
Flat forehead and face	+	+	+
Scaphocephaly	+	+	+
Uplifted earlobes	-	-	-
Broad nasal bridge	-	-	-
Rounded nasal tip	-	-	-
Prominent columella	-	-	-
Medial flaring of the eyebrows	-	-	-
Large eyebrows	-	-	-
Sparse eyebrows in the middle	-	-	-
Open mouth	+	-	+
Microcephaly	+ at birth	NA	+
M-shaped upper lip	-	-	-
Deep-set eyes	-	-	-
Central depression of the earlobes	-	-	-
Strabismus	-	-	-
Epicanthus	-	-	-
**Hypotonia**	+	NA	-
**Musculoskeletal anomalies**	+short hands and broad fingers	NA	-
**Short stature**	IUGR	NA	+
**Neurodevelopment delay**	+	+	+
**Seizures**	-	NA	-
**Brain structural anomalies**	-	NA	+
**Congenital heart defects**	+small atrial septal defect	+ToF, patent foramen ovale	+atrial septal defect
**Abdominal abnormalities**	+ constipation	NA	+ hernia, constipation
**Urogenital anomalies**	+small testes	NA	-
**Dental anomalies**	+teeth dysplasia	NA	+ intermittent spaced teeth
**Behavioral problems**	+happy, sociable, timid	NA	+crybaby
**Other features**	-	hypernasal voice	-

*IUGR* Intrauterine growth retardation, *TOF* Tetralogy of Fallot, *NA *Not available

Contiguous gene gain syndrome including entire *ZEB2* characterized with similar multiple congenital anomalies of MWS and the distinctive craniofacial features is mainly caused by large 2q22 repeats including *ZEB2* leading to dominant singe *ZEB2* gene gain mutation, which is recommended to be named “Liu-Liang-Chung” syndrome. We diagnose this novel syndrome to distinguish it from MWS.

## Discussion and conclusions

Liu-Liang-Chung syndrome is a novel syndrome. To date, only 3 cases including our patient, with data of detailed clinical features of the syndrome, have been reported. These patients all have large 2q22 repeats including *ZEB2* leading to dominant singe *ZEB2* gene gain mutation. CNVs of these cases are summarized in Supplemental Table 1. These cases present neurodevelopment retardation, congenital heart disease, constipation, dental anomalies, behavioral problems, inguinal hernia, and urogenital anomalies, which are also common features in MWS patients. However, none of these individuals has the typical facial features of MWS, who all have approximately same distinctive craniofacial features that are entirely different from MWS. Contiguous gene gain syndrome including entire *ZEB2* is a complex chromosome abnormal syndrome, so the novel syndrome we named can better distinguish Mowat–Wilson syndrome to avoid diagnostic confusion like two different syndromes with different anomalies in the same 22q11.2 region, such as DiGeorge syndrome (chromosome 22q11.2 deletion syndrome) and chromosome 22q11.2 duplication syndrome. Baxter et al. reported a case of MWS caused by a partial duplication of *ZEB2*, encompassing exons 1 and 2 as well as intron 1 and part of intron 2 [11]. Therefore, a partial duplication of *ZEB2* should not be the pathogenesis of Liu-Liang-Chung syndrome, but, the increase of whole *ZEB2* gene dosage is genetic etiology of the syndrome.

*ZEB2* gene (zinc finger E-box protein 2, also named as Smad interacting protein 1, *SIP1*) encodes a protein that is a member of the δEF1/ Zfh-1 family of two handed zinc finger/homeodomain transcription factors included in the TGF-β/BMP/Smad-mediated signalling cascade [6, 12]. SIP1 protein may exert a lot of important roles in embryogenesis process [12–14]. *SIP1* mRNA was detected with high levels in almost all adult human tissues (heart, brain, placenta, lung, liver, squeletal muscle) as well as in mouse heart and brain [6]. *SIP1* gene is ubiquitous expressed in diencephalon, mesencephalon, rhombencephalon, spinal cord, cranial nerve ganglia (V, VII, IX, X), spinal ganglia, sympathetic ganglia, neurectoderm, neural retina, anterior epithelium of the lens, foregut, midgut, hindgut (ganglia and mesenchyma), genital tubercle, perichondrum, myotoma, mesonephros (surrounding tissue), metanephros (mesenchyme), mesenchyme in early human embryo [15]. There were also findings that overexpression of Xenopus *SIP1* induced enlargement of neural tissue in anterior region and some embryos failed to form eye vesicles [16]. *ZEB2* as a dosage sensitive gene, *ZEB2* copy number gain is functionally and clinically significant at 2q22.3 involving triplication of *ZEB2* gene [9]. The whole *ZEB2* dosage increase in Liu-Liang-Chung syndrome may cause multiple system abnormalities like MWS, but in a different way, the distinct craniofacial features that are entirely different from MWS may be generated, while other genes can play subtle regulatory roles, which create other phenotypic differences such as growth and development retardation, protruding ears and so on.

Individuals with 2q23.1 microduplication syndrome including either all or a portion of methyl-CpG-binding domain 5 (*MBD5*) gene, display phenotype including intellectual disability, language impairments, infantile hypotonia and gross motor delay, behavioral problems, autistic features, dysmorphic facial features (pinnae anomalies, arched eyebrows, prominent nose, small chin, thin upper lip, et al.), and minor digital anomalies (fifth finger clinodactyly and large broad first toe) [17]. A significant overlap of craniofacial features including eyes, thin upper lip, micrognathia may be shown between our case and individuals with the 2q23.1 microduplication syndrome, however, our case has more phenotypes such as congenital heart disease and constipation, no prominent nose and wide mouth, which reveal that *ZEB2* is more pathogenic than *MBD5* by an increase in gene dosage at 2q21.2-q24.1. In chromosomal region of dosage sensitive genes at 2q, there may be more cis-acting elements that affect subsequent gene expression of multiple systems by increase of dosage, and at the same time, abnormal dosages of key sensitive genes also play the precise regulating roles in each section deleteriously.

In summary, we report a novel Liu-Liang-Chung syndrome with a novel *de novo* 22.16 Mb duplication at 2q21.2-q24.1. Among phenotypic characteristics of the syndrome, the distinctive craniofacial features can be more easily recognized, which are first reported as a guide for precise clinical and genetic diagnosis of the syndrome. Some variable additional features in the syndrome including remarkable growth and development retardation and protruding ears were recognized for the first time.

### Supplementary Information

**Additional file 1:** **Supplemental Fig. 1.** Imaging findings of the present case. **Supplemental Fig. 2.** CNVs showing A 22.16 Mb duplication. **Supplemental Fig. 3.** Fq-PCR showing A 22.16 Mb duplication. **Supplemental Table 1.** CNVs of cases with Liu-Liang-Chung syndrome. 

## Data Availability

The datasets generated and/or analysed during the current study are included in this article.

## Abbreviations

MWS: Mowat–Wilson syndrome
*ZEB2*: Zinc finger E-box protein 2
EEG: Electroencephalogram
MRI: Magnetic resonance imaging
NGS: next‑generation sequencing
CNVs: Chromosomal copy-number variations
*SIP1*: Smad interacting protein 1
*MBD5*: Methyl-CpG-binding domain 5
