# Supplementary material for: “Liu-Liang-Chung” syndrome with multiple congenital anomalies and the distinctive craniofacial features caused by dominant ZEB2 gene gain mutation
Source: BMC Pediatr. 2023 Sep 21;23:480. doi: 10.1186/s12887-023-04314-5 (PMC10512491; doi:10.1186/s12887-023-04314-5)
Supplement: Supplementary file 1 — Additional file 1: Supplemental Fig. 1. Imaging findings of the present case. Supplemental Fig. 2. CNVs showing A 22.16 Mb duplication. Supplemental Fig. 3. Fq-PCR showing A 22.16 Mb duplication. Supplemental Table 1. CNVs of cases with Liu-Liang-Chung syndrome. [file 12887_2023_4314_MOESM1_ESM.docx]

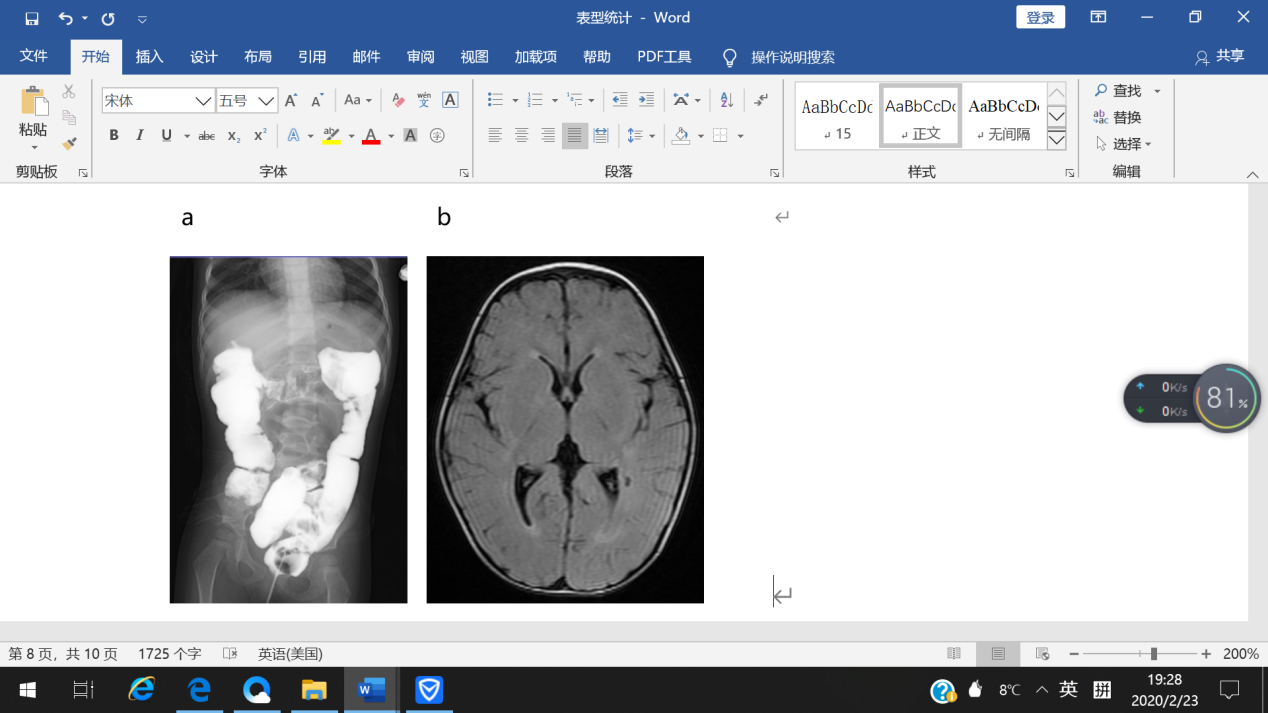


Supplemental Figure 1. Imaging findings of the present case. (a) Abdominal radiology of barium enema, (b) Brain MRI.


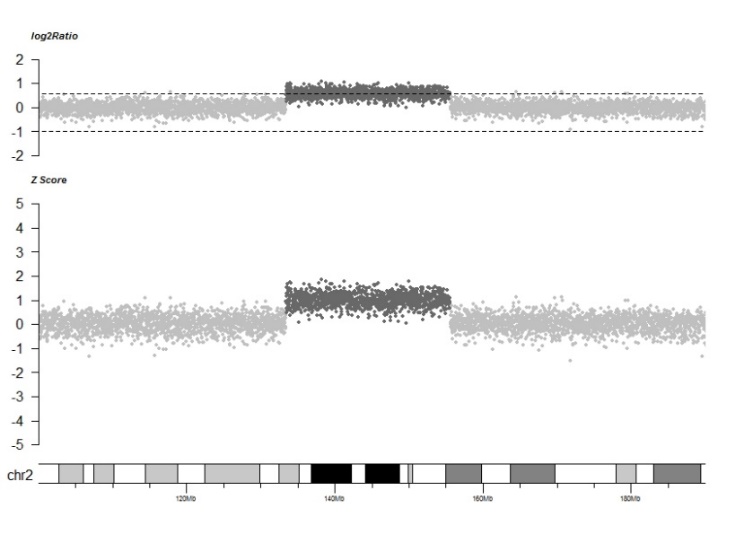


Supplemental Figure 2. CNVs showing A 22.16 Mb duplication. A 22.16 Mb duplication at 2q21.2-q24.1, (133,460,001- 155,620,000) x3, was showed using CNVs in our case (shown in dark black).

Supplemental Figure 3. Fq-PCR showing A 22.16 Mb duplication. A 22.16 Mb duplication at 2q21.2-q24.1, (133,460,001- 155,620,000) x3, was identified using fq-PCR in our case, as assessed by *NCKAP5*, *LRP1B*, *KCNJ3*, *ZEB2* genes at 2q21.2-q24.1 compared with a normal control *GAPDH* gene times of 1.40-1.6.

**Supplemental Table1**. CNVs of cases with Liu-Liang-Chung syndrome

| Case (reference) | Case1 [9] | Case 2 [10] | Our case |
| --- | --- | --- | --- |
| Size | 2.9 Mb | 2.1Mb | 22.16 Mb |
| Inheritance | *de novo* | *de novo* | *de novo* |
| Chromosomal location | 2q22.2q22.3  143,886,436–146,831,592 | 2q22.3  144,223,416-  146,233,601 | 2q21.2-q24.1  133,460,001-155,620,000 |
| Type | duplication(chr2:143,886,436-144,391,185),  triplication(chr2:144,391,186-146,831,592) | duplication | duplication |
| Genes contained | *ARHGAP15, GTDC1, ZEB2, TEX41* | *ARHGAP15, GTDC1, ZEB2* | *NCKAP5*, *MGAT5*, *TMEM163*, *ACMSD*, *CCNT2*, *RAB3GAP1*, *ZRANB3*, *R3HDM1*, *MIR128-1, UBXN4*, *LCT*, *MCM6*, *DARS1*, *CXCR4*, *HNMT*, *NXPH2*, *LRP1B*, *KYNU*, *ARHGAP15*, *GTDC1*, *ZEB2*, *ACVR2A*, *ORC4*, *MBD5*, *EPC2*, *KIF5C*, *LYPD6*, *MMADHC*, *RND3*, *NMI*, *TNFAIP6*, *RIF1*, *NEB*, *ARL5A*, *CACNB4*, *STAM2*, *FMNL2*, *PRPF40A*, *ARL6IP6*, *RPRM*, *GALNT13*, *KCNJ3* |
